# *In silico* prediction of targets for anti-angiogenesis and their *in vitro* evaluation confirm the involvement of SOD3 in angiogenesis

**DOI:** 10.18632/oncotarget.24693

**Published:** 2018-04-03

**Authors:** Javier A. García-Vilas, Ian Morilla, Anibal Bueno, Beatriz Martínez-Poveda, Miguel Ángel Medina, Juan A.G. Ranea

**Affiliations:** ^1^ Universidad de Málaga, Andalucía Tech, Departamento de Biología Molecular y Bioquímica, Facultad de Ciencias, And IBIMA (Biomedical Research Institute of Málaga), Málaga, Spain; ^2^ CIBER de Enfermedades Raras (CIBERER), Málaga, Spain

**Keywords:** angiogenesis, matrigel assay, predictive networks, superoxide dismutase 3, wound scratch assay

## Abstract

Biocomputational network approaches are being successfully applied to predict and extract previously unknown information of novel molecular components of biological systems. In the present work, we have used this approach to predict new potential targets of anti-angiogenic therapies. For experimental validation of predictions, we made use of two *in vitro* assays related to two key steps of the angiogenic process, namely, endothelial cell migration and formation of “tubular-like” structures on Matrigel. From 7 predicted candidates, experimental tests clearly show that superoxide dismutase 3 silencing or blocking with specific antibodies inhibit both key steps of angiogenesis. This experimental validation was further confirmed with additional *in vitro* assays showing that superoxide dismutase 3 blocking produces inhibitory effects on the capacity of endothelial cells to form “tubular-like” structure within type I collagen matrix, to adhere to elastin-coated plates and to invade a Matrigel layer. Furthermore, angiogenesis was also inhibited in the *en vivo* aortic ring assay and in the *in vivo* mouse Matrigel plug assay. Therefore, superoxide dismutase 3 is confirmed as a putative target for anti-angiogenic therapy.

## INTRODUCTION

An important goal in the field of protein interactions is to recognise interactions that are involved in disease genesis and progression. The majority of the protein interaction space is still unchartered and hidden to scientists. In spite of the efforts devoted to elucidate interactomes in the last decades, surprisingly only about 10% of human protein-protein interactions (PPIs) are experimentally known, about one-third of human proteins have no known interactions, and more than half of all identified human protein-coding genes have no experimental evidence of their function [1, 2]. The exploration of these unknown areas of proteins functionality has been enhanced with the emergence of new high-throughput techniques. Unfortunately, assay complexity and costs make genome-scale knockdown experiments unaffordable for many research groups. Additionally, the search of novel targets frequently implies extensive experimental screenings and further investigation of literature and databases. The arduous task, the cost and the risk involved in the study of new targets in many cases deter researchers to initiate such investigations.

These limitations can bias the research towards the over-investigation of a bunch of already well characterized targets [3]. This trend is also present in the search of new drug therapies by the industry, producing a large set of drugs organized around a privileged set of few druggable domains [4]. As an example, in angiogenesis-dependent diseases such as cancer, therapy is mainly focused on the inhibition of different members of the family of vascular endothelial growth factors (VEGFs) and their receptors. Despite the important role of VEGF pathway in the angiogenic process and the consequent initial optimism about the VEGF-targeting therapy in the treatment of cancer, its effectivity only has been demonstrated in a limited number of cancer types, as colorectal cancer, due in part to the mechanisms of resistance observed in the majority of tumors. The implication of multiple pathways in the regulation of angiogenesis and the redundancy in the vessel formation mechanism determine the complexity of the process and evidence the emergence of compensatory mechanisms when a single target is inhibited [5–7]. The existance of different strategies to inhibit angiogenesis, implemented as monotherapy or in a combinatory approach, is a good marker of this intrinsic complexity [8]. In line with this, much more effort is required for the identification of novel anti-angiogenic targets that could represent new and more effective alternative therapies to the already used treatments.

In this work we have carried out the search of novel anti-angiogenic targets using computational means as an efficient methodology to select a manageable set of candidate genes for further experimental validation. We wanted to make use of PPI knowledge to predict novel candidate targets for inhibition of angiogenesis, a crucial process for tumor progression and metastasis as well as for many angiogenesis-dependent diseases [9, 10]. By exploiting the context information present in large PPI networks, some computational methods have shown to be useful for the efficient selection, in whole genomes, of small sets of candidate genes associated to different functional systems [3, 11–14]. In this work we applied such computational methods to search for potential novel anti-angiogenic targets, based on the physical and functional relationships of these candidates to a set of already known members of the angiogenic protein system through the whole human interactome.

To measure functional relationship within large interaction networks between novel candidates and known angiogenic proteins, in the present work we have implemented a graph kernel based on the random walk-with-restart (RWR) algorithm as described by us elsewhere [15]. The same predictor algorithm based on kernels has been extensively used and validated in the selection of candidate genes in collaboration with others [3, 16]. This method generates ranked lists of candidate genes [17], in this case related to the human angiogenic process, integrating all the connection information between proteins and avoiding local topological artifacts in PPI neworks that depend on highly connected proteins or hubs. Seven selected candidate targets for angiogenesis inhibition were experimentally evaluated *in vitro* by testing the effects of their gene silencing or antibody blocking on two key steps of the angiogenic process, namely, endothelial cell migration and “differentiation” in tubular-like structures on Matrigel [18–20]. As a result of this validation, extracellular isoform of superoxide dismutase (ecSOD or SOD3) emerges as a putative new target for anti-angiogenic therapy, showing promising results inhibiting endothelial cell migration and “differentiation” *in vitro* and preventing angiogenesis *ex vivo* and *in vivo*. This secreted oxidoreductase catalyzes the dismutation of superoxide anion to hydrogen peroxide and its importance in cellular defense against oxidative stress has been widely described [21]. In addidtion, a role for SOD3 in neovascularization after ischemic injury through modulation of VEGF signaling has been described [22, 23]. In view of the results showed in this work, we propose SOD3 as a novel target for therapy in angiogenesis-dependent diseases such as cancer.

## RESULTS

### Predictions and selection for experimental testing

**Figure 1:**
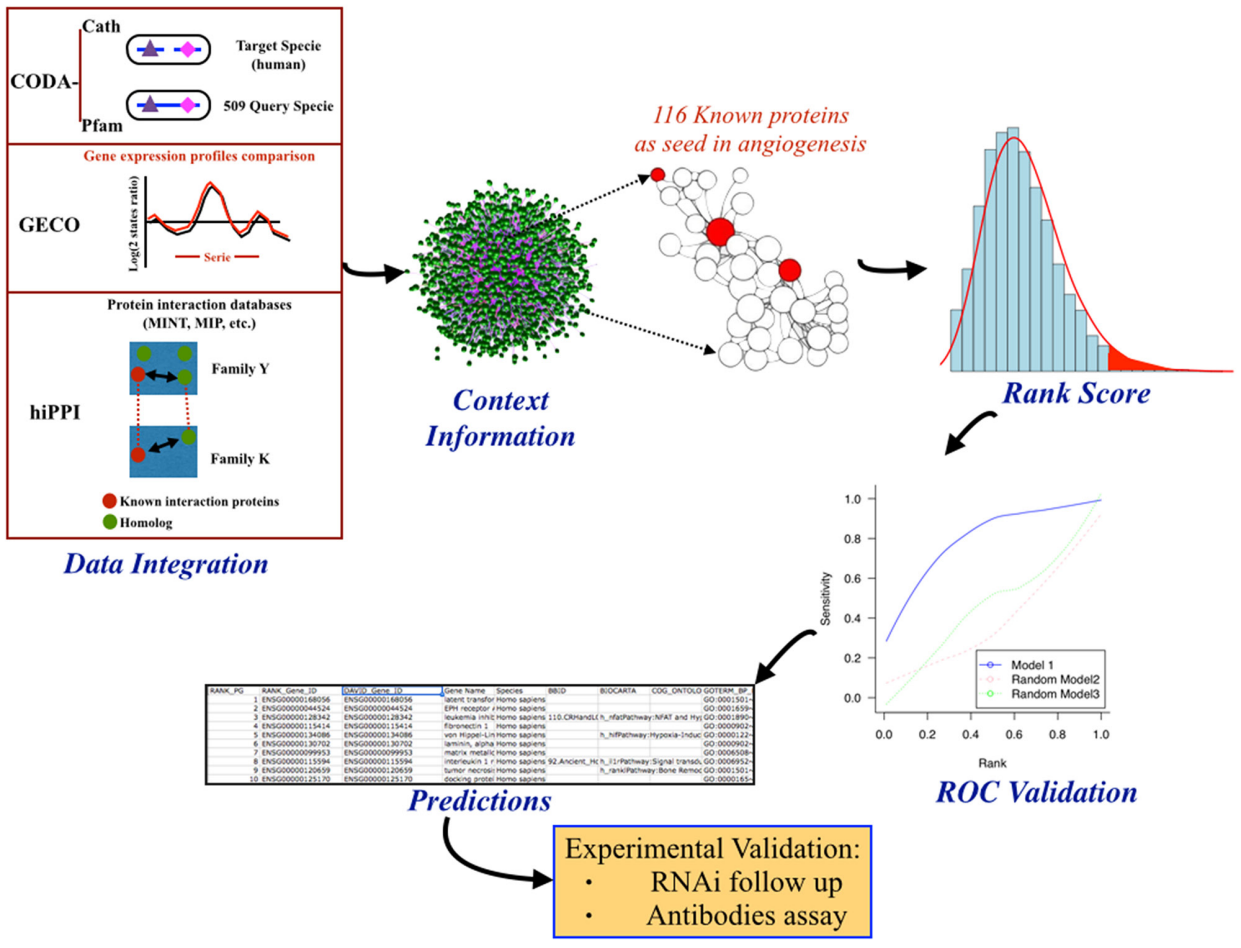
General pipeline followed in this work with description of the steps followed in the initial predictive biocomputational analysis Data integration of protein-protein interaction (PPI) predictions from domains co-occurrence data (CODA), gene expression similarity (GECO), interaction protein homology relationships (hiPPI) and other PPI datasets into one single PPI network model. This human interactome model is used to implement context information analyses by computing kernel distance scores between target proteins and a seed set of 116 known angiogenic proteins. These kernel scores are used to rank target proteins in a priorization list that is validated using a leave-one-out cross-validation method represented by the ROC validation curve. Finally, a set of target proteins are selected from the top ranked list of predictions for their further experimental validation.

The biocomputational procedure summarized in Figure 1 included data integration, extraction of new information based in the context, random walk kernel, rank scoring and ROC curve validation, giving finally rise to a ranked list of predicted putative new gene products involved in angiogenesis. The application of this procedure on the whole human interactome yielded a total ranked list of 19,618 human proteins. Candidate proteins with a closer relationship to the greater number of known angiogenic protein dataset, in the context of interactions of the human PPI network, appear at the top of the ranked list. Therefore, we focused for a detailed further study on the top ranked three hundred statistically most promising proteins. These top ranked proteins were filtered by an expert manual curation based on available literature and biological databases in order to confirm their novelty as angiogenic proteins. Later on, the pre-selected novel promising candidates were filtered again based on their experimental handling feasibility, considering the possibility of designing specific siRNA against their transcripts and the commercial availability of specific antibodies at affordable costs. Finally a set of seven genes were selected for their further experimental validation (see Table 1).

**Table 1:** List of pre-selected and selected predicted (highlighted in fold) genes putatively involved in angiogenesis to be experimentally tested

*RANK_PG*	*DAVID_Gene_ID*	*Gene Name(Symbol)*
35	ENSG00000109047	Recoverin (*RCVRN*)
47	ENSG00000174611	Kyphoscoliosis peptidase (*KY*)
49	ENSG00000134453	RNA binding motif protein17 (*RBM17*)
**50**	**ENSG00000109814**	**UDP-glucose 6-dehydrogenase (*UGDH*)**
56	ENSG00000182208	MOB kinase activator 2 (*MOB*)
60	ENSG00000137076	talin 1 (*TLN1*)
69	ENSG00000101346	Protein O-fucosyltransferase1 (*POFUT1*)
70	ENSG00000116459	ATP synthase peripheral stalk-membrane subunit b *(ATB5PB*)
**144**	**ENSG00000135914**	**5-hydroxytryptamine receptor 2B (*HTR2B*)**
**190**	**ENSG00000124440**	**hypoxia inducible factor 3 alpha subunit (*HIF3A*)**
214	ENSG00000163754	glycogenin 1 (*GYG1*)
**217**	**ENSG00000139973**	**synaptotagmin 16 (*SYT16*)**
218	ENSG00000143469	synaptotagmin 14 *(SYT14*)
229	ENSG00000182985	cell adhesion molecule 1 (*CADM1*)
**242**	**ENSG00000122729**	**aconitase 1 (*ACO1*)**
**258**	**ENSG00000109610**	**superoxide dismutase 3 (*SOD3*)**
**276**	**ENSG00000134824**	**fatty acid desaturase 2 (*FADS2*)**
277	ENSG00000149485	fatty acid desaturase 1 (*FADS1*)
278	ENSG00000221968	fatty acid desaturase 3 (*FADS3*)

### Silencing of selected putative targets

To test the selected genes as targets for anti-angiogenesis, we analyzed the effects of either siRNA silencing or gene product blocking with specific antibodies on two key steps of angiogenesis, namely, endothelial cell migration and “differentiation” to “tubular-like” structures on Matrigel.

**Figure 2:**
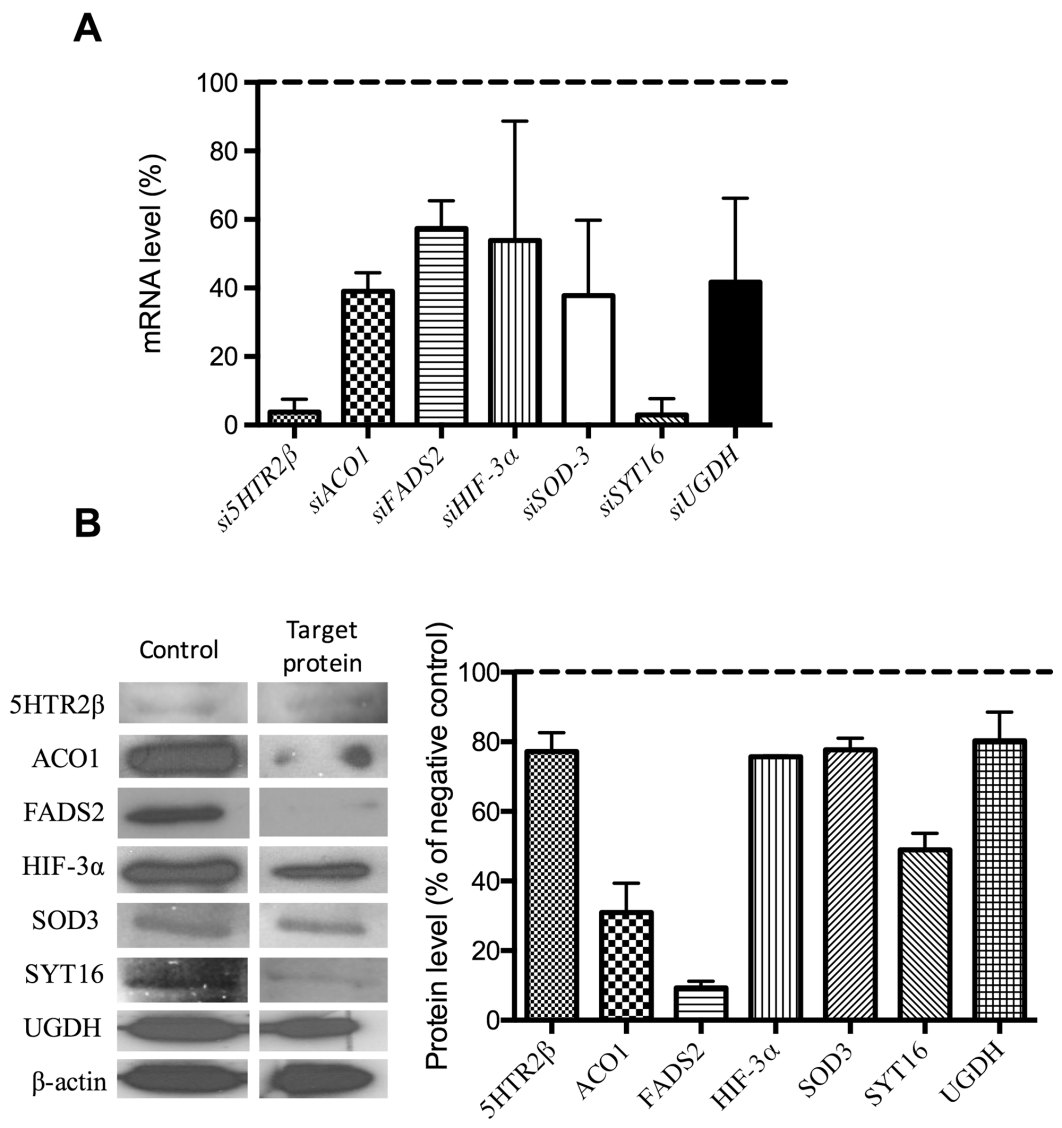
Checking of gene silencing **(A)** mRNA expression levels of target genes in endothelial cells treated with specific siRNA. **(B)** Representative images and quantification of protein levels corresponding to target genes in endothelial cells treated with specific siRNA. All the shown quantitative data exhibited statistically significant differences with values for control, untreated samples (p<0.05). Data represent the means ± SD of three independent experiments.

Silencing was carried out with the siRNAs listed in Table 2. For each silencing treatment, both mRNA and protein levels were determined by qPCR (with the primers and conditions listed in Table 3) and Western blotting (with the antibodies listed in Table 4), respectively. Figure 2 shows that all the silencing treatment yielded significant reductions in both the mRNA (Figure 2A) and protein (Figure 2B) levels. Only siHTR2 and siSYT16 decreased serotonin receptor 2-beta (HTR2) and synaptotagmin XVI (SYT16) mRNA levels by more than 90%. In contrast, their protein levels decreased only by 22% and 50%, respectively. The rest of silencing treatments yielded only decreases between 40 and 60% in mRNA levels (Figure 2A). Only fatty acid desaturase 2 (FADS2) and aconitase 1 (ACO1) protein levels were strongly reduced by more than 90 and 70%, respectively (Figure 2B).

**Table 2:** List of siRNAs

	ID esiRNA	Gene name	Organism
Sigma-Aldrich	HU-06353-1	*ACO1*	Human
Sigma-Aldrich	HU-16048-1	*FADS2*	Human
Sigma-Aldrich	HU-06710-1	*HIF3a*	Human
Sigma-Aldrich	HU-05288-1	*HTR2B*	Human
Sigma-Aldrich	HU-05295-1	*UGDH*	Human
Sigma-Aldrich	HU-03535-1	*SYT16*	Human
Applied Biosystems	S13272	*SOD3*	Human

**Table 3:** List of primers and conditions used in qPCR for quantification of mRNAs

Gene	Sequence Primer	Annealing Temperature	Amplicon Size
*HTR2B*	Forward: 5´ggatgcggttaaaagagaa 3´ Reverse: 5´ ccaattgccctcttgacaat 3´	65°C	102 bp
*FADS2*	Forward: 5´ aatcagcaggggtttcaaga 3´ Reverse: 5´ ggcactacgctggagaagat 3´	65°C	92 bp
*HIF-3α*	Forward: 5´ gaatgggtctgcgagagtgt 3 Reverse: 5´ cccagtcggagagtatcgtc 3´	63°C	108 bp
*UGDH*	Forward: 5´ ttcctcgacaggattctacca 3´ Reverse: 5´ cagggtaacggttgttgatgt 3´	60°C	110 bp
*ACO1*	Forward: 5´caatggctcagcaaggtgt 3´ Reverse: 5´ctgcttgggtcaggttcg 3´	64°C	108 bp
*SYT16*	Forward: 5´cgccagagctgttggtgggg 3´ Reverse: 5´tgggctgaccacgccgaatg 3´	60°C	192 bp
*SOD3*	Forward: 5´cttctgtattctgctattggttattc 3´ Reverse: 5´atgctgttctatgccccattctg 3´	60°C	183 bp

**Table 4:** List of antibodies

	Antibody	Dilution
GenWay	ACO1	1/200
AbCam	FADS2	1/1000
AbCam	HIF3ɑ	1/500
AbCam	HTR2B	1/1000
AbCam	UGDH	1/1000
AbCam	SOD3	1/1000
AbCam	SYT16	1/500

**Figure 3:**
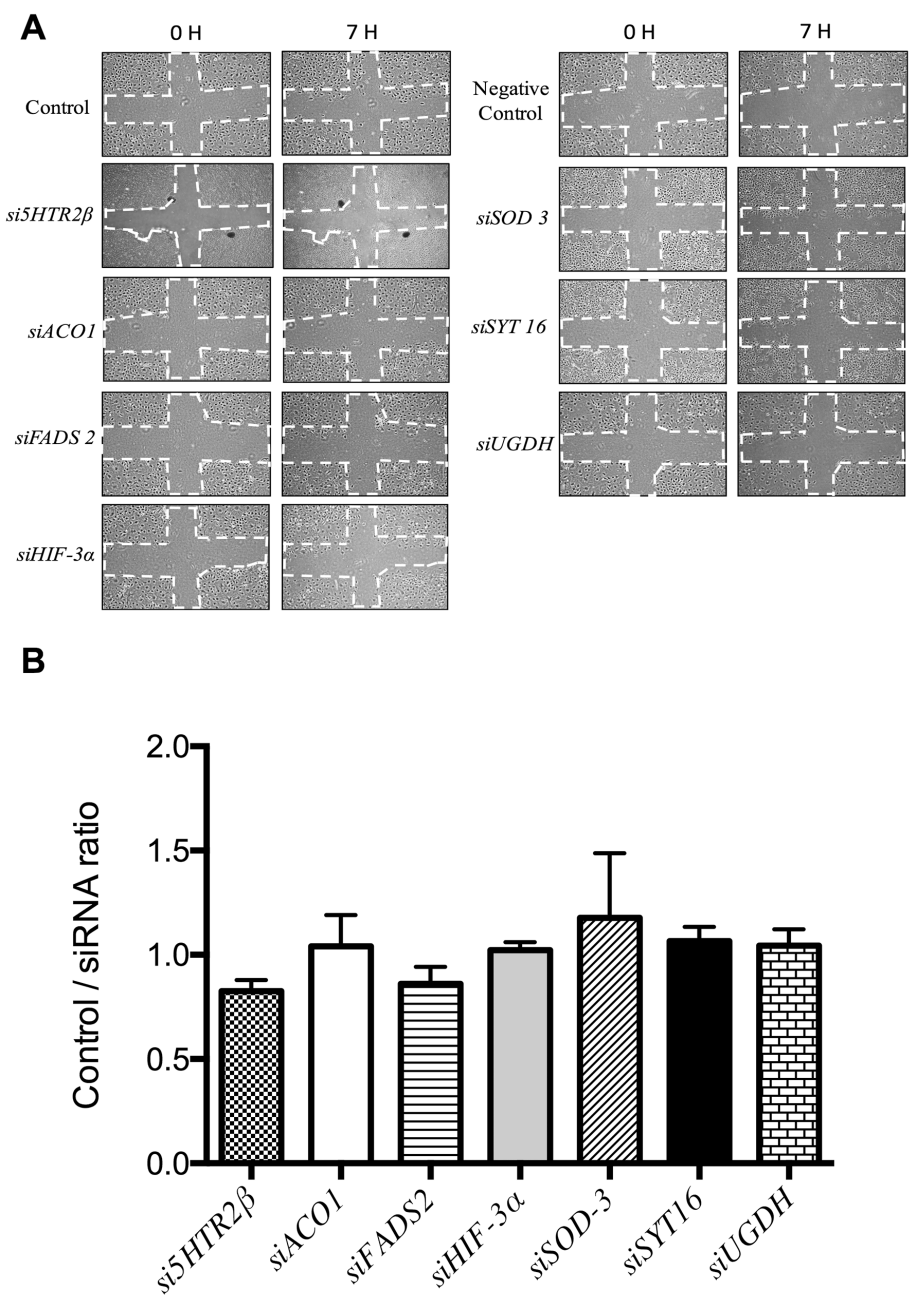
Wound healing assay to analyze endothelial cell migration **(A)** Representative photographs taken at time 0 and 7 h. The dashed lines show the initial area without cells. Controls are untreated HMEC endothelial cells and “negative controls” are cells with only the transfection reagents added. **(B)** Quantitative analysis of wound healing data for HMEC cells. Data represent the means ± SD of three independent experiments and they are given as ratios (dividing the signals in control samples by the corresponding siRNA experiment signals).

**Figure 4:**
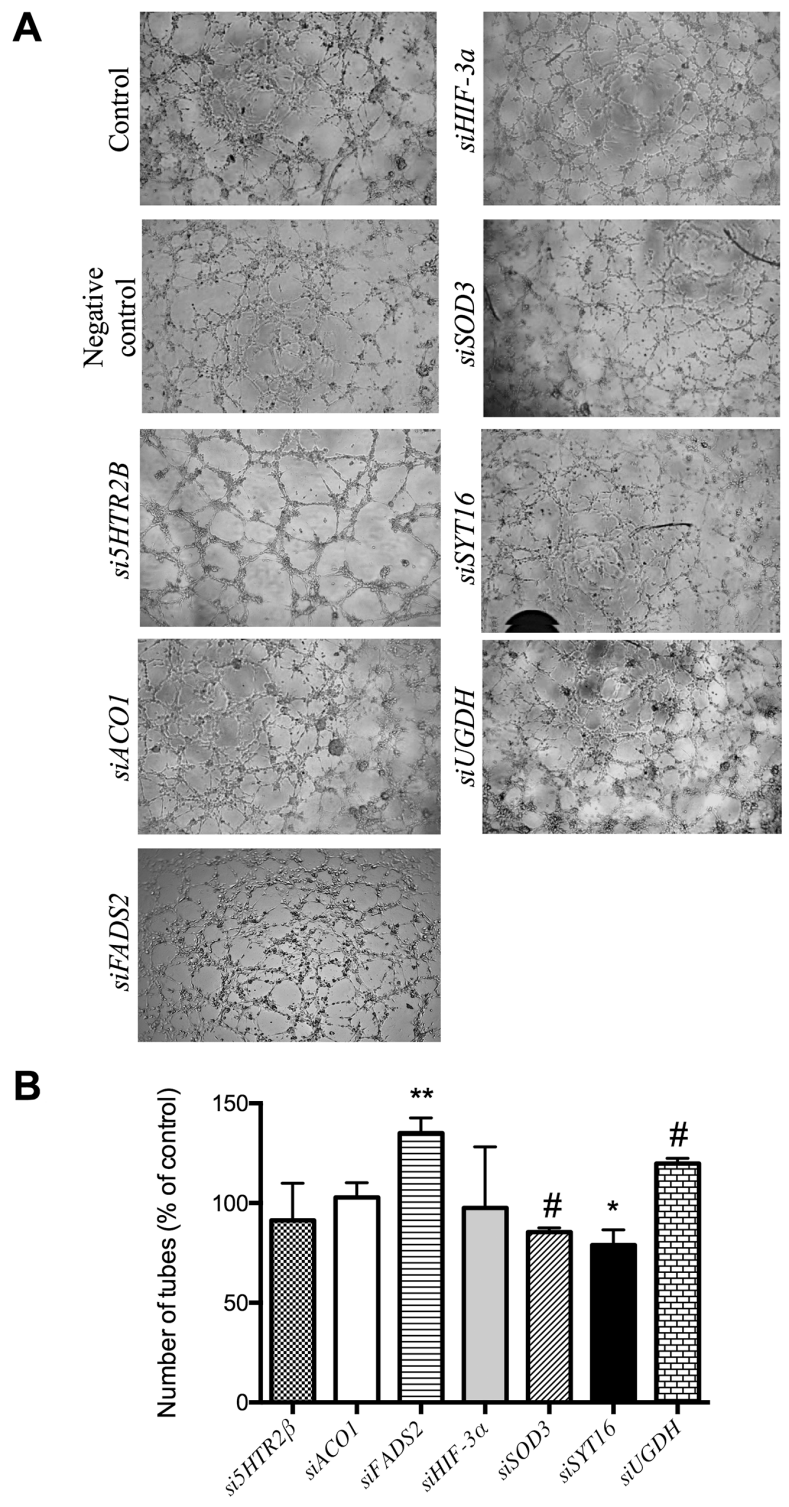
Formation of “tubule-like” structures of endothelial cells on Matrigel **(A)** Representative photographs of control and gene-silencing treated cells on Matrigel after 5 h. **(B)** Quantitative analysis of data. Data represent the means ± SD of three independent experiments. Symbols indicate significant differences between control and treated cells (^*^p <0.05, ^**^p<0.01, ^#^p < 0.005).

Figure 3 shows that no silencing treatment caused any significant effect on the mobility of endothelial cells as tested with the wound healing assay. Figure 4 shows that only siSOD3 and siSYT16 significantly reduced the number of “tubular-like” structures formed by endothelial cells grown on Matrigel. It is noteworthy that silencing treatment of *FADS2*, which yielded more than 90% of reduction in FADS2 protein levels (Figure 2B), significantly increased the number of “tubular-like” structures formed on Matrigel as compared with control, untreated endothelial cells (Figure 4). There was also a slight increase in the number of “tubular-like” structures in the case of siUGDH treatment.

In any case, it should be stressed that none of the silencing treatments produced complete inhibitory effects on the two *in vitro* assays of angiogenesis used in the present study.

### Blocking of selected putative targets with specific antibodies

**Figure 5:**
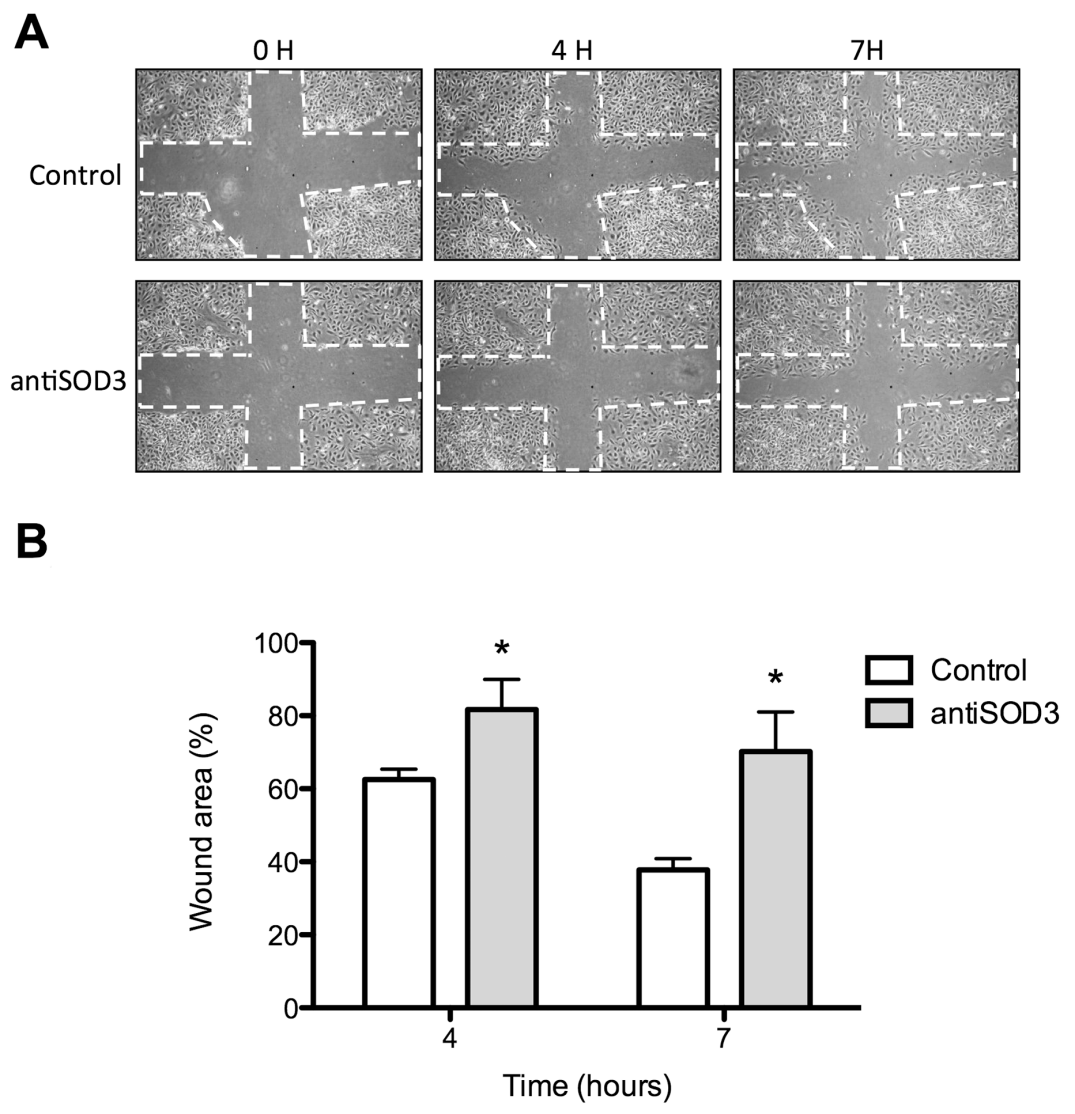
Wound healing assay to analyze migration of endothelial cells treated with antiSOD3 **(A)** Representative photographs of control and antiSOD3-treated cells in the wound healing assay at different times. The dashed lines show the initial area without cells. **(B)** Quantitative analysis of data. Data represent the means ± SD of three independent experiments. Symbols indicate significant differences between control and treated cells (^*^p <0.05).

**Figure 6:**
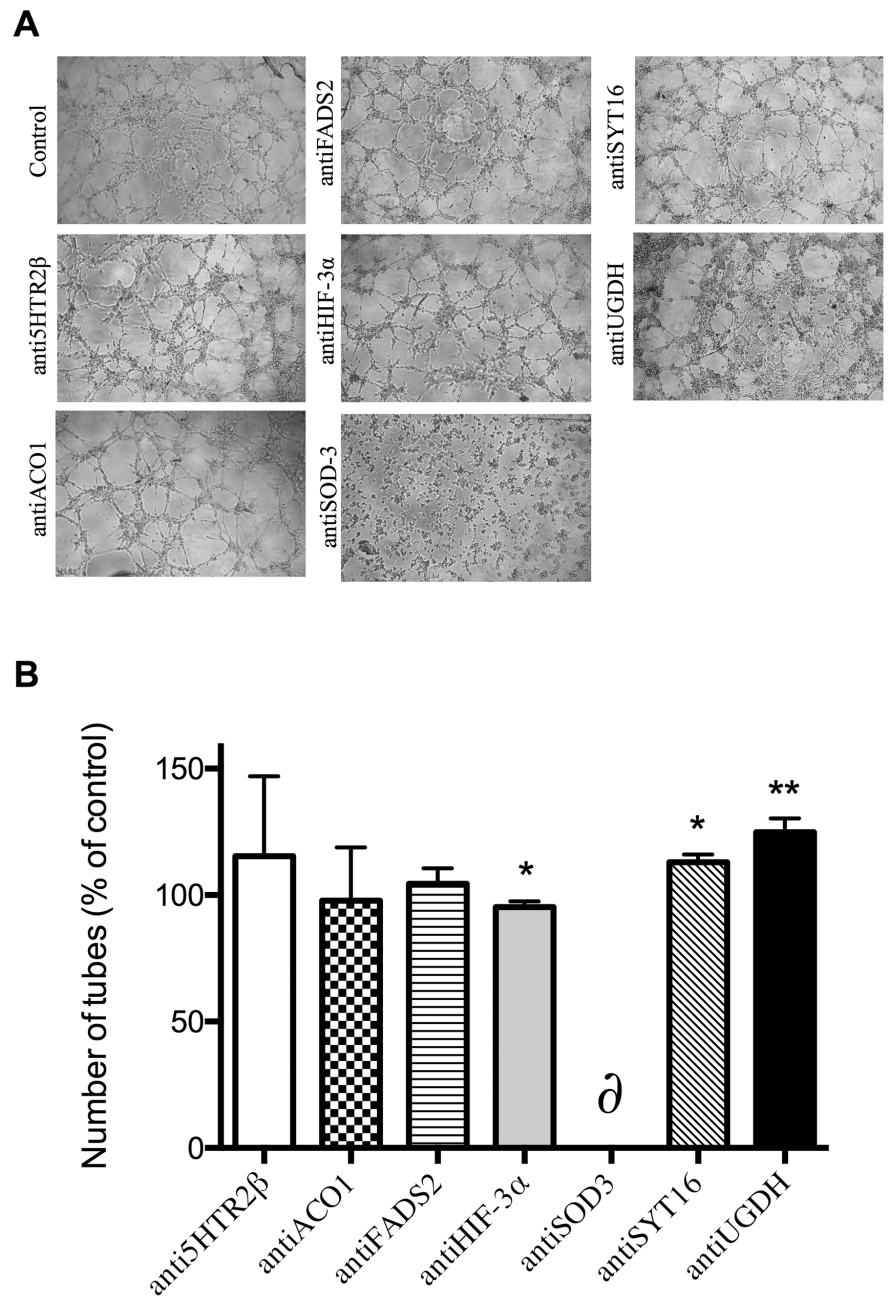
Formation of “tubule-like” structures of endothelial cells on Matrigel **(A)** Representative photographs of control and antibody-treated endothelial cells on Matrigel after 5 h. **(B)** Quantitative analysis of data. Data represent the means ± SD of three independent experiments. Symbols indicate significant differences between control and treated cells (^*^p <0.05, ^**^p<0.01). The δ symbol means a complete inhibition of tube formation in three different, independent experiments for the case of antiSOD3 treatment.

The use of specific antibodies to block the selected putative targets yielded more relevant effects than silencing treatment. Figure 5 shows that antiSOD3 significantly decreased the endothelial cell migratory potential. No other tested antibody treatment produced any effect on the migratory potential of endothelial cells as determined by the wound healing assay (results not shown). On the other hand, Figure 6 shows that antiHIF-3ɑ significantly decreased and antiSYT16 and antiUGDH significantly increased the number of tubes formed by endothelial cells grown on Matrigel. Nonetheless, the most relevant result is the complete inhibition of “tubular-like” structures formation with antiSOD3.

### *In vitro* and *ex vivo* assays confirm that blocking of SOD3 with specific antibodies inhibits angiogenesis

**Figure 7:**
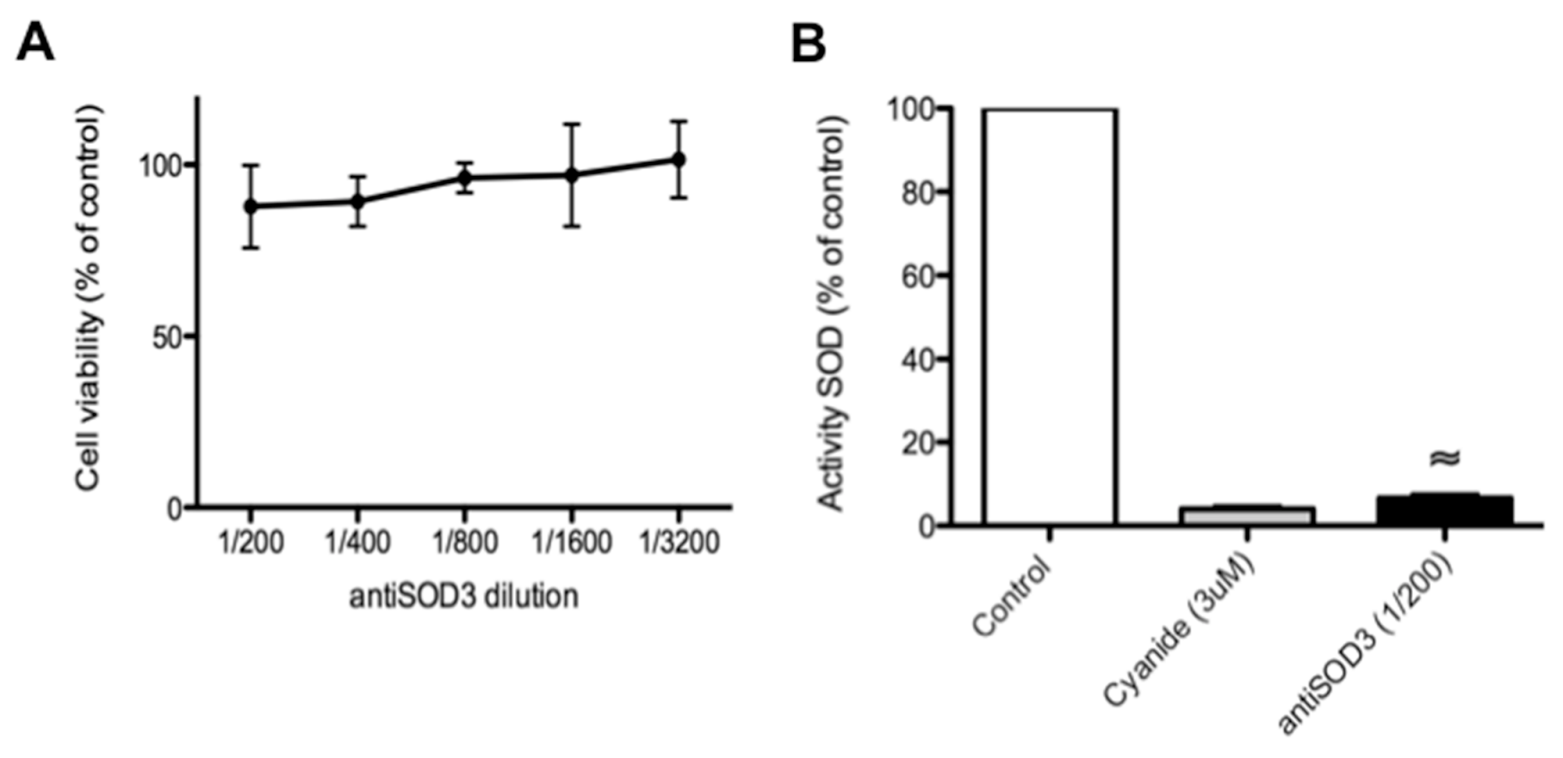
Blocking of SOD3 with antiSOD3 does not affect endothelial cell viability but inhibits *in vitro* SOD activity **(A)** Effect of antiSOD3 specific antibody on HMEC viability after 24 h of incubation. Data are given as means ± S.D. of three independent measurements and they are expressed as percentages taking as 100% the cells in controls incubated in the absence of antibody. **(B)** *In vitro* determination of SOD activity in the presence or absence of antiSOD3 specific antibody. As a positive control of SOD inhibition, we determined SOD activity in the presence of 3 μM cyanide. Data represent the means ± SD of three independent measurements and they are expressed as percentages taking as 100% the SOD activity in controls incubated in the absence of antibody. The symbol indicates significant differences between control and treatment (p < 0.0005).

According to the whole set of results presented above, the most promising predicted target seemed to be SOD3. For this reason, next we centered our attention on the effects of antiSOD3 in several additional *in vitro* and *ex vivo* angiogenesis assays. However, we carried out some control experiments before showing that antiSOD3 had no significant effects on endothelial cell survival after 24 h of treatment (Figure 7A) and strongly inhibited *in vitro* SOD activity (Figure 7B).

**Figure 8:**
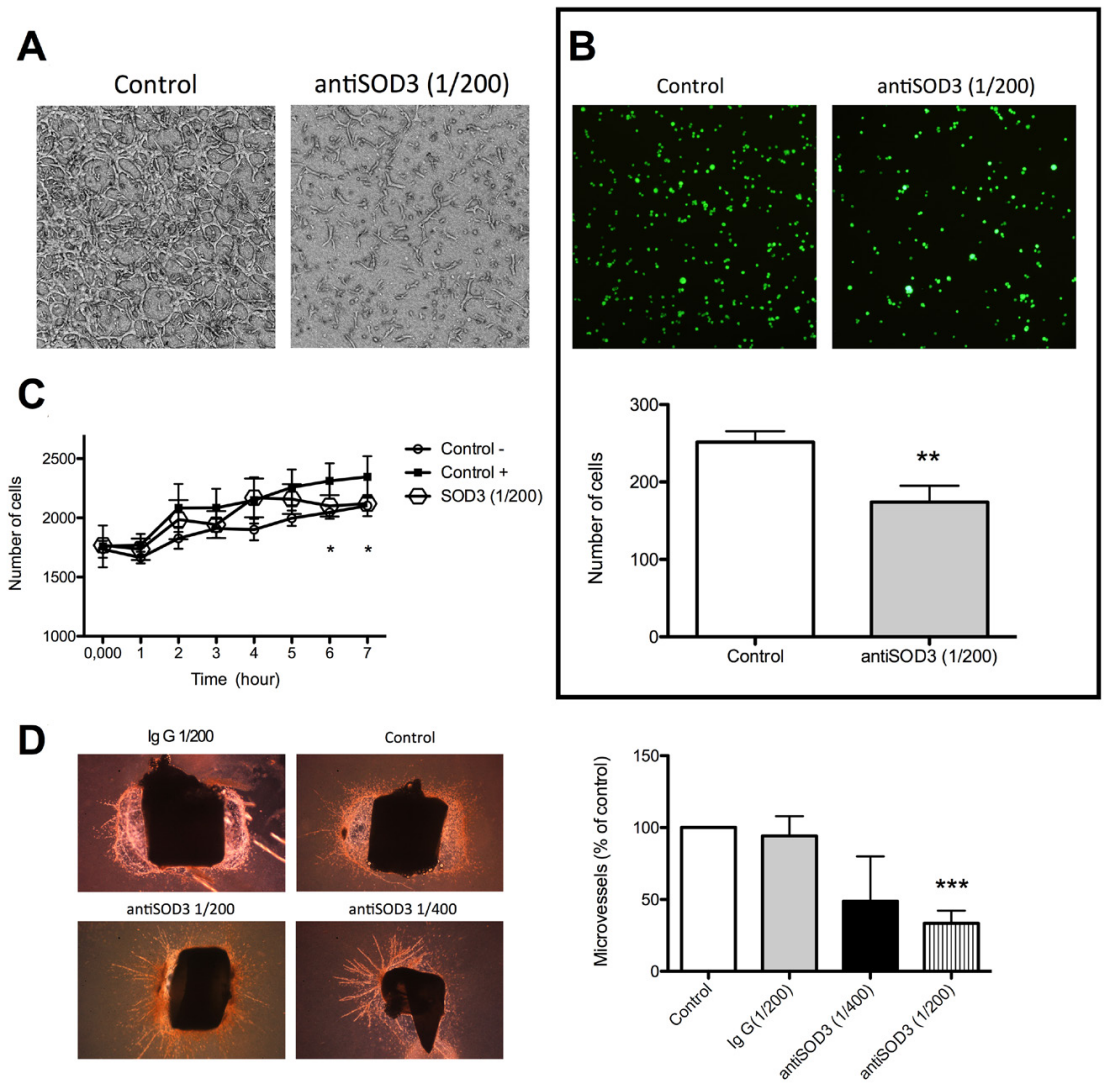
Blocking of SOD3 with antiSOD3 inhibits *in vitro* and *ex vivo* angiogenesis **(A)** Inhibition of “tubular-like” structures within a 3D matrix. Representative photographs of control and antibody-treated endothelial cells within a 3D type I collagen matrix. **(B)** Inhibition of endothelial cells to elastin. Representative photographs of control and antibody-treated, calcein-labeled endothelial cells adhered to elastin and quantitative analysis of data. **(C)** Quantification of increasing number of invading cells with time in the invasion assay. **(D)** Inhibition of *ex vivo* angiogenesis. Representative photographs of control and antibody-treated rat aortic ring explants after 14 days of culture and quantitative analysis of data. Data represent the means ± SD of three independent experiments. Symbols indicate significant differences between control and treated cells (^*^p <0.05, ^**^p<0.01, ^***^p<0.005).

To confirm that the blockage of SOD3 with specific antibodies inhibited tubulogenesis *in vitro*, we checked the formation of “tubular-like” structures within a 3D collagen matrix. Figure 8A shows that blocking SOD3 completely inhibited tubulogenesis in this assay. Furthemore, we studied if antiSOD3 treatment resulted in a decrease of adhesiveness of HMEC, since this is a critical characteristic of endotelial cells to migrate and to reorganizate into “tubular-like” structures. Adhesion assay showed that the adhesiveness of HMEC to elastin *in vitro* was partially but significantly inhibited by SOD3 blocking (Figure 8B). The effect of antiSOD3 treatment on invasive capacity of endothelial cells was assessed using a transwell assay (Figure 8C). Blockage of SOD3 reduces the invasión of HMEC significantly at 6-7 hours after the start of the assay, in correlation with the data obtained in migration assay (Figure 5). In view of *in vitro* data, we decided to perform an *ex vivo* angiogenesis assay, namely rat aortic ring assay. We studied two dilutions of antiSOD3 antibody (1/400 and 1/200) in this assay and we quantified the formation of microvessels in the different conditions (Figure 8D). At the 1/200 antiSOD3 dilution, microvessels density in explants was significantly decreased compared to the IgG control explants, showing that blockage of SOD3 reduced sprouting from aortic rings *ex vivo*.

### Blocking of SOD3 with specific antibodies clearly inhibits angiogenesis *in vivo*

**Figure 9:**
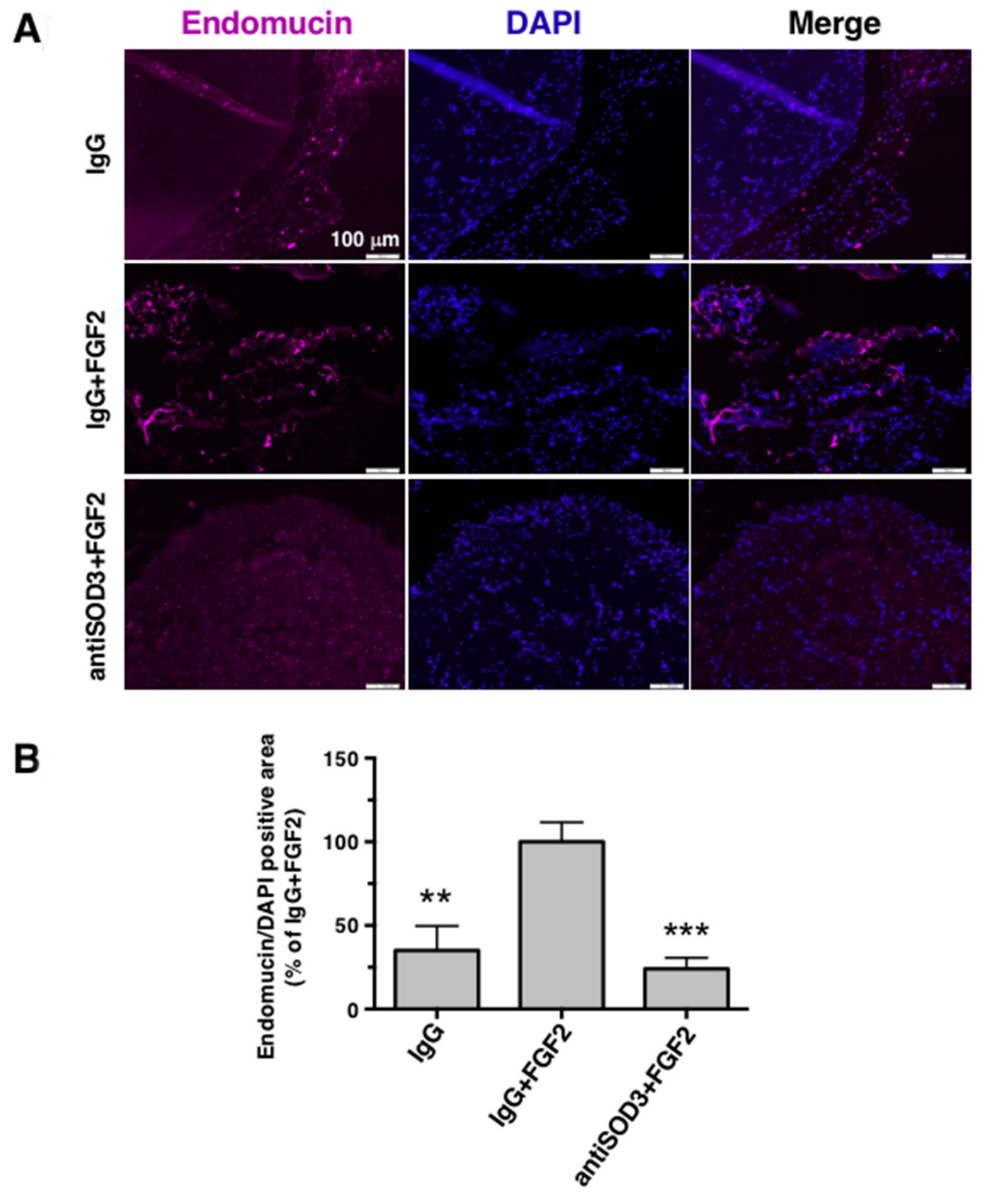
Blocking of SOD3 with antiSOD3 inhibits *in vivo* angiogenesis in the mouse Matrigel plug assay **(A)** Inhibition of *in vivo* angiogenesis. Paraffin sections of Matrigel plugs with different conditions (IgG, IgG+FGF2 or antiSOD3+FGF2) were stained with anti-endomucin (endothelial marker) and DAPI (nuclear marker). Representative pictures at 10X magnification are showed. Scale bars represent 100 μm. **(B)** Endomucin positive areas were quantified using ImageJ software, relativized to total DAPI signal, and all data were expressed as means ± SD of 3 plugs (IgG) or 4 plugs (rest of conditions) and normalized to the IgG+FGF2 condition (100%). Symbols indicate significant differences between IgG+FGF2 and rest of conditions (^**^p<0.01, ^***^p<0.005) using a Student’s t test.

The promising results obtained from *in vitro* and *ex vivo* experiments prompted us to perform a mouse *in vivo* angiogenesis plug assay. Comparing with IgG+FGF2 containing plugs (positive control of vascularization), antiSOD3+FGF2 containing plugs showed a clear reduction in vessels presence, similar to the vascularization level obtained in IgG plugs (negative control of vascularization) (Figure 9A and 9B). Of note, the inhibition of angiogenesis in this assay was even higher than the quantification shown in (Figure 9B), since pictures without signal for endomucin presented a very high background due to microscope setting normalization.

## DISCUSSION

Our predictive network approach has previously been shown to be useful to extract new information from the “dark matter” of biological data accumulated in databases [1]. When we applied the general pipeline depicted in Figure 1, we were able to extract apparently new information pointing to potentially new targets for anti-angiogenesis. Nonetheless, it should be underscored that for at least 4 of the 7 selected predicted putative “new” gene products involved in angiogenesis we could find out in literature previous claims of experimental data suggesting their involvement in angiogenesis. This is the case of UDP-glucose dehydrogenase (UGDH), serotonin receptor 2 beta (HTR2B), hypoxia inducible factor 3 alpha (HIF-3ɑ) and superoxide dismutase 3 (SOD3). UGDH inhibition has been claimed to diminish tumor angiogenesis *in vivo* [24]. However, in this case the claim refers to a previous article that does not mention UGDH at all [25]. Selective inhibition of HTR2B with SB204741 has been shown to reduce microvessel densitity in tumors [26]. Regarding SOD3 (also known as extracellular superoxide dismutase), it was shown several years ago to have an essential role in reparative neovascularization induced by hindlimb ischemia [22]. On the other hand, since HIF-1ɑ is a master regulator of angiogenesis, a potential involvement of HIF-3ɑ in angiogenesis could also be proposed. However, a recent identification and characterization of the HIF-3ɑ transcriptional program has found out that neither VEGF nor VEGF pathway were affected by this transcription factor [27].

Taking our experimental results altogether, only one of the 7 selected putative targets for anti-angiogenesis (SOD3) has proven its potential unambiguously. Although SOD3 silencing only decreased SOD3 protein levels by 22%, this decrease was enough to yield a mild but significant inhibitory effect in the Matrigel tubulogenesis assay (Figure 4), pointing to the importance of SOD3 in this process. Accordingly, blocking of SOD3 protein with its specific antibody yielded clearer and more potent effects, with a significant decrease of endothelial cell migration (Figure 5) and a complete inhibition of endothelial cell formation of “tubular-like” structures on Matrigel (Figure 6). Furthermore, in the present study we have shown that SOD3 blocking also produces a complete inhibition of tubular-like structure formation by HMEC cultured within a type I collagen matrix (Figure 8A). It is also known that SOD3 can interact with some extracellular matrix proteins. This is the case of fibulin-5, a component of extracellular matrix that contributes to the formation of elastic fibers and is strongly expressed in the embryonic vasculature and in injured adult vessels [28]. Moreover, SOD3 co-locates with type I collagen, thus protecting it against oxidative fragmentation [29]. In the present work, we have shown that HMEC treated with anti-SOD3 decrease their adhesiveness capacity to elastin (Figure 8B). The angiogenic phenotype of endothelial cells also involves their potential to invade spaces occupied by extracellular matrix. Our results also support a role for SOD3 on endothelial cell invasive potential, since SOD3 blocking decreased it (Figure 8C). Taken altogether, our *in vitro* results support a pro-angiogenic role for SOD3 and identify it as a promising target for anti-angiogenesis. This suggestion is strongly reinforced by our results in the *ex vivo* aortic ring assay (Figure 8D), showing that anti-SOD3 decreased the number of new vessels sprouting from the aortic ring after 14 days of incubation.

SOD enzymes are oxidoreductases that catalyze the dismutation of superoxide anion to hydrogen peroxide and their importance in cellular defense against oxidative stress has been widely described [21]. Currently a vascular protective role is accepted for superoxide dismutase isoforms expressed in the vessel wall [30]. In particular, it has been shown that high amount of SOD3, the extracelular isoform, localized between the endothelium and the smooth muscle layer of the vessels, and its presence preserves vascular function in ageing, in hypertension and in heart failure models [21]. On the other hand, SOD3 has been shown to regulate blood flow and pressure [31, 32]. SOD3 has also been related to inhibition of inflammation, as well as to protection against chronic obstructive pulmonary disease and against renal ischemia/reperfusion injury [33–36]. Regarding the connections of SOD3 with angiogenesis, it has also been shown that SOD3 is able to preserve lung angiogenesis in neonatal mice after hyperoxia exposure [37]. In addition, it has been shown that SOD3 expression is markedly increased in bone marrow after ischemia and that SOD3 has an essential role in reparative angiogenesis induced by ischemia [22]. The mechanism of action of SOD3-induced vascularization after ischemic events involves the activation of VEGF-receptor type2 signaling via H_2_O_2_-mediated oxydative inactivation of protein tyrosine phospatases (PTPs) in caveolae/lipid rafts microdomains [23]. These data are in agreement with an overall pro-angiogenic role for SOD3. In contrast, it has been shown that SOD3 overexpression leads to suppression of the hypoxic accumulation of HIF-1ɑ in human pancreatic cancer cells, with a concomitant decrease in VEGF levels [38]. Taken together, our results support a pro-angiogenic role for SOD3 and identify it as a promising target for anti-angiogenic therapy in cancer and other angiogenesis-related diseases.

The exact mechanism of action by which SOD3 blockage inhibits migration of endothelial cells and tubular-like structures formation *in vitro*, and angiogenesis *ex vivo* and *in vivo* remains to be elucidated. Although the described role of this enzyme in the activation of VEGFR2 points to VEGF pathway as the main responsible for the pro-angiogenic activity of SOD3 [23], our *in vivo* results in the FGF2-driven angiogenesis plug assay suggest that SOD3 could be modulating not only VEGF signaling but other angiogenic pathways such as FGF2/FGFR. The activation of several compensatory pathways have been proposed upon VEGF/VEGFR blocking approaches, explaining the tumor refractoriness observed in anti-angiogenic therapies [39]. It remains to be determined if SOD3 is implicated in the activation of not only VEGF pathway, since in other compensatory pathways such as FGF signaling, given the role of this enzyme as a key enzyme in the regulation of the extracellular oxidative stress. Future studies on endothelial cell lines will clarify this point.

Previous reports about the role of SOD3 in revascularization processes after ischemic event [22] together with the promising results presented in this work clearly point to SOD3 as a potential new target for anti-angiogenesis. Despite this, currently there is an absence of annotation of SOD3 as angiogenic related protein in public databases, and therefore *SOD3* is not recognized as angiogenic-related gene in any extense analysis of the human proteome. In view of the information available, we propose to include the angiogenesis-related implication of SOD3 in public databases.

Regarding the rest of the putative targets selected by the predictive biocomputational network approach, experimental assays rejected the direct involvement of HTR2B, ACO1, and SYT16 on endothelial cell migration and differentiation. The partial effects caused by *FADS2*, *HIF-3*ɑ and *UGDH* silencing and by the blocking of their respective proteins are unconclusive and they deserve to be further analyzed in the future.

## CONCLUDING REMARKS

**Figure 10:**
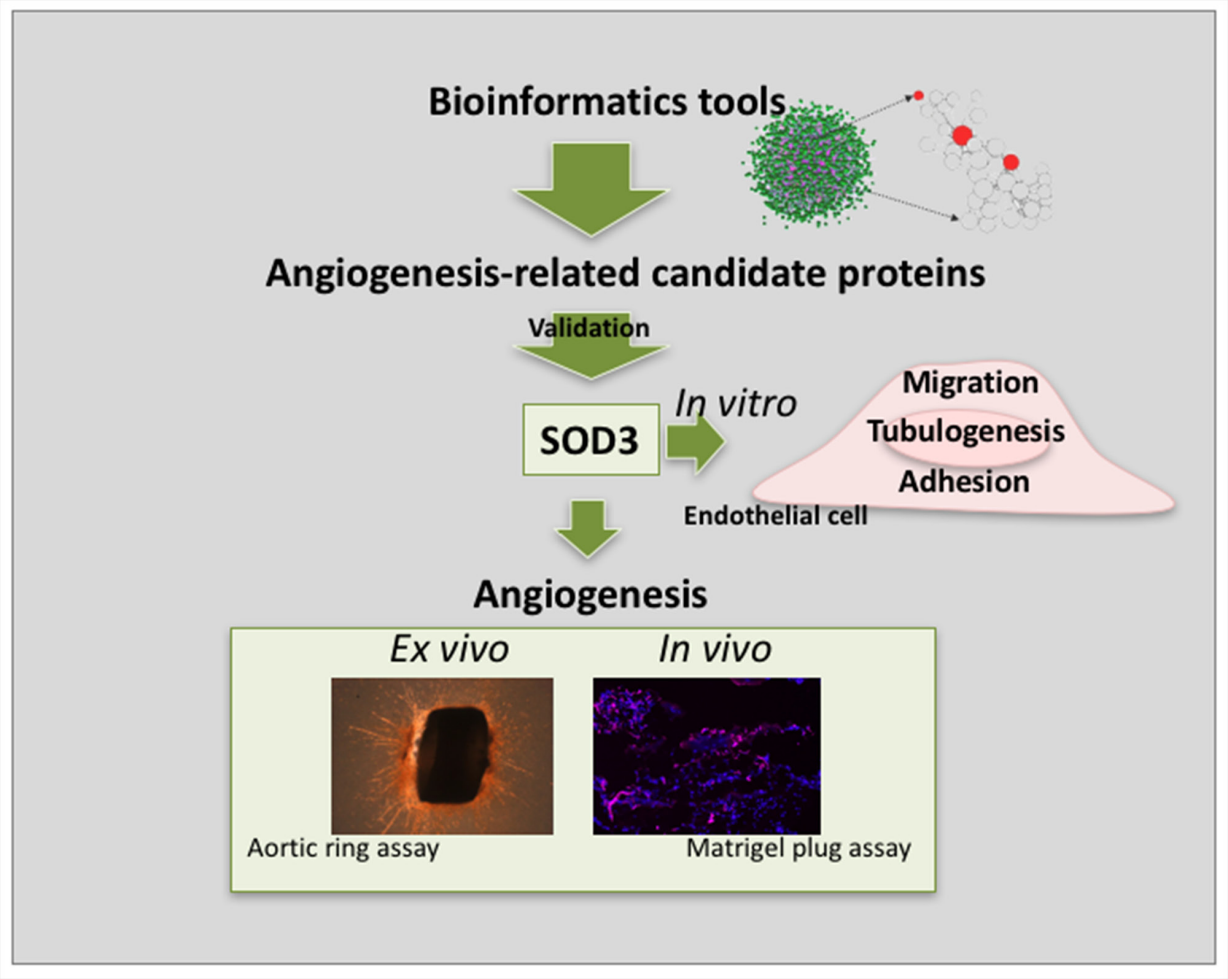
Schematic summary showing principal findings of the work The implementation of bioinformatics tools allowed the identification of a number of angiogenesis-related candidate proteins. After curation of the results and functional validation, SOD3 emerged as a protein potentially implicated in angiogenesis. *in vitro*, *ex vivo* and *in vivo* results presented in this work point to SOD3 as a novel anti-angiogenic target.

Our predictive, biocomputational network approach has proven to be able to extract useful information from biological “dark matter” [1]. This study confirms the involvement of SOD3 in angiogenesis showing that SOD3 blocking completely abrogates endothelial cell “differentiation” and partially inhibits endothelial cell migration, two key steps of the angiogenic process. Moreover, our *in vivo* results clearly shows that blocking of SOD3 activity prevents angiogenesis. Altogether, our results point to SOD3 as a promising target for anti-angiogenesis (see the summarizing scheme in Figure 10). It is important to stress that none of the selected candidate genes are classified as angiogenic related protein in public databases. This absence of annotation was proved in a comprehensive search in tens of public repositories using DAVID web server tool (see Supplementary Table 1 in Supplementary Material) [40, 41]. Therefore, these genes would not be recognized as angiogenic-related genes in any extense analysis of the human proteome.

## MATERIALS AND METHODS

### Algorithm and data sources for biocomputational predictions

Figure 1 summarizes the general pipeline followed in this work for biocomputational predictions. The data processing steps, biological networks construction and algorithm used by this work have been described by us elsewhere [3, 16]. In brief, we efficiently computed a kernel (random walker with restart –RWR- algorithm) to weigh the functional relationships between proteins on large PPI networks resulted from the integration of various human PPI data sources as previously described: protein domain co-occurrence data, gene expression similarity, and interacting proteins homology relationships (Figure 1). Other public sources of protein-protein association data were also used for comparison, such as: Reactome, Kegg, Intact, MINT and HRPD databases. Measures of functional relationships were calculated using RWR between each candidate gene and a set of 116 human proteins known to be related to the angiogenesis process [42]. The method performance was validated using a leave-one-out cross-validation method represented by the ROC curves described by us elsewhere [3, 16]. The method ranks candidate proteins based on the global weight of their association to the 116 angiogenic protein dataset used as a seed.

### Chemicals and reagents

Supplements and other chemicals not listed in this section were obtained from Sigma Chemicals Co. (St. Louis MO, USA). Cell culture media, penicillin, streptomycin and amphotericin B were purchased from Biowhittaker (Walkersville, MD, USA). Fetal bovine serum (FBS) was a product of Harlan-Seralb (Belton, United Kingdom). Human serum (HS) was a product of Lonza (Basel, Switzerland). Plastics for cell culture were supplied by NUNC (Roskilde, Denmark) and VWR (West Chester, Pennsylvania, USA). Small interference RNA (siRNA) section were obtained from Sigma Chemicals Co. (St. Louis MO, USA). Antibodies used in this work were purchased from AbCam (Danvers, MA, USA). HiPerfect Transfection Reagent was purchased from Qiagen (Hilden, Germany).

### Cell cultures

The immortalized human dermal microvascular endothelial cell (HMEC) line kindly supplied by Dr. Arjan W. Griffioen (Maastrich University, The Netherlands) was used in this study. This cell line has been previously characterized [43]. Cells were grown in RPMI-1640 medium supplemented with glutamine (2 mM), penicillin (50 IU/mL), streptomycin (50 mg/L), amphotericin (1.25 mg/L), 10% fetal bovine serum, and 10% human serum. The predicted targets selected for experimental analysis were submitted to silencing of their mRNA expression (see next section) or blocking of the gene products by using the specific antibodies listed in Table 4.

### Transient transfections

For gene silencing, HMEC cells were grown in 6-well plate and transfected with 50 nM MISSION^®^esiRNA (Sigma-Aldrich) using Hi-Perfect-Transfection reagent (Qiagen) as described by manufacturer´s instructions. Table 2 contains the list of siRNAs used in this work.

### Quantitative RT-PCR

For quantitative RT-PCR (qPCR), total RNA isolation and complementary DNA synthesis were performed as described previously [44] and PCR reactions were done using KAPA SYBR Fast Master Mix (2x) Universal (KAPA Biosystems) in a EcoTM Real- Time PCR System. qPCR was performed in triplicate for each sample according to the manufacturer’s instructions. All qPCR data were normalized to HPRT expression. Primers, amplicon size, and qPCR conditions for each gene are shown in Table 3.

### Western blotting

For Western blotting, treated (both silenced with siRNA and blocked with antibodies) and control cells were lysed in Laemmli’s loading buffer 2X and boiled for 5 min at 95°C; samples were separated in a SDS-PAGE electrophoresis and blotted onto a nitrocellulose membrane. After blocking in TBS-T plus 5% w/v dry non-fat milk, membranes were probed with primary antibodies overnight at 4°C. Membranes were washed in TBS-T and probed with secondary antibody linked to horseradish. peroxidase for 1 hour at room temperature. After washing, membranes were developed using the ECLTM system (Amersham Biosciences) or the SuperSignal West Pico Chemiluminiscent Substrate system (Thermo).

### Scratch wounding migration assay

The migratory activity of endothelial cells was assessed using a “wound-healing” migration assay. Confluent monolayers in 6-well plates were wounded with pipet tips following two perpendicular diameters, giving rise to two acellular 1-mm-wide lanes per well. After washing, treated and control cells were supplied with 1.5 mL of complete medium. Photographs were taken at different times of incubation (after 4 and 7 h). The amount of migration at these time points was determined by image analysis (ImageJ software) in both control and treated wells.

### Tubulogenesis assays on matrigel and within 3D collagen matrix

In the 2D assay, a total of 5·10^4^ cells (both control and treated ones) were added with 200 μL of medium. Cells were incubated at 37°C in a humidified chamber with 5% CO_2_. After 5 h of incubation, cultures were observed and photographed with a Nikon inverted microscope DIAPHOT-TMD (Nikon Corp., Tokyo, Japan). Each concentration was tested in triplicate. “Tubular”structures were counted using image analysis tools. Each coherent, noninterrupted, closed network was counted as a “tube”.

In the 3D assay, 5×10^5^ HMEC cells were seeded in complete medium in pre-coated 6-well plates. These plates were coated with 1 mL of a mix 1:1 of collagen 2 mg/mL and derived HBBS dissolution (HBBS 10x, NaHCO_3_, NaOH, H_2_O, penicillin/streptomycin and L-glutamine). The antibody SOD3 was added on cells and was incubated 5 h at 37°C in a humidified 5% CO_2_ atmosphere. After that, 1 mL of the same mentioned mix was left on the subconfluent culture and, after polymerization, 2 mL of RPMI-1640 medium with 2% of FBS was added over this matrix. The formation of capillary-like structures after 72 h of incubation was photographed (Nikon DS-Ri2) with an inverted and phase-contrast microscope (Nikon Eclipse Ti).

### MTT Cell viability assay

The 3-(4, 5-dimethylthiazol-2-yl)-2-5-diphenyltretrazolium bromide (MTT) dye reduction assay in 96-well microplates was used. The assay is dependent on the reduction of MTT by mitochondrial dehydrogenases of viable cells to a blue formazan product, which can be measured spectrophotometrically. Endothelial cells (2.5 × 10^3^ cells in a total volumen of 100 μL of complete medium) were incubated in each well with serial dilutions of SOD3 antibody. After 1 h of incubation in the dark (37°C, 5% CO_2_ in a humid atmosphere), 10μL of MTT (5 mg/mL in PBS) was added to each well, and the plate was incubated for further 4 h (37°C). The formazan was disolved in 150 μL of 0.04 N HCl-2 propanol, and samples were spectrophotometrically measured at 550 nm. All determinations were carried out in quadruplicate, and at least three independent experiments were carried out.

### Superoxide dismutase activity

Superoxide dismutase activity was determined through the rate of cytochrome C reduction measured spectrophotometrically at 550 nm. The quantification was done in buffer mix (50 mM potassium phosphate buffer, 0.1 mM ethylene diamine tetraacetic acid, 0.01 mM cytochrome C, 0.05 mM xanthine solution, and 0.005 units of xanthine oxidase). 10 units/mL of superoxide dismutase was diluted in cold purified water. Superoxide dismutase enzyme solution was mixed with SOD3 antibody (at a final dilution of 1/200) and it was incubated for 30 minutes at 25°C. Buffer mix was added to the superoxide dismutase enzyme solution with SOD3 antibody and the final mix was incubated for 5 minutes at 25°C. Finally, the rate of cytochrome C reduction was spectrophotometrically measured at 550 nm.

### Adhesion assay

The wells of 24-well plates were covered with 300 μL of 10 μg/mL elastin and maintained at 4°C overnight. Then, the leftover ungelled elastin was removed, 300 μL of 7.5% bovine serum albumin was added to each well, and plates were maintained at 37°C for another hour, and afterwards they were withdrawn. Endothelial cells were incubated at 37°C in the absence o presence of the tested dilution of SOD3 antibody for 24 h. Calcein-AM (1 mg/mL) was added to cells 2 h prior the end of incubation. After incubation, cells were washed twice with PBS. Cells were suspended at 3 × 10^5^ cells/mL in complete culture medium, and 300 μL of cell suspension was added to each well. After 1 h of incubation at 37°C, wells were gently washed three times with PBS. Finally, cells that remained attached were counted and photographed in an inverted epifluorescence Nikon microscope.

### Cell invasion assay using transwells

Invasion of fluorescence-labeled cells was assayed by using membrane insert in 24-well fluorescence-opaque plates. This assay allows for a real-time monitoring of the process because it eliminates the need to remove non-invading cells before quantifying invading cells. HMEC cells were grown to 80-90% confluence in RPMI-1640 medium and labeled *in situ* with 5 μg/mL Calcein-AM in complete culture medium for 2 h at 37°C. After washing, the cell monoloyer was briefly trypsinized to lift the cells, which were washed and suspended in culture medium with 0.1% BSA. Cells were added to 8 μm FALCON HITS FluoroBlok inserts (Becton Dickinson, Bedford, MA) at a density of 2 × 10^5^ cells/insert in the absence or presence of SOD3 antibody. Filters of inserts were previously coated with Matrigel (25 μL/filter) for their use in the invasion assay. RPMI-1640 medium with 10% FBS and 10% HS was used as a chemoattractant in the lower wells. The inserts were incubated at 37°C and the real time kinetics of cell invasion were determined by taking readings at differents times with a Fluorescence Microplate Reader (FL600FA, BIO-TEK Instruments, Winooski, VT, USA) in the bottom read mode using excitation/emission wavelengths of 485/530 nm and a gain setting of 75. Relative velocities of invasion for control and treated cells were compared.

### Rat aortic ring assay

Thoracic aortas were removed from 12 weeks old Wistar rats and immediately transferred to a culture dish containing DMEM. The perioaortic fibroadipodise tissue was carefully removed with fine microdissecting forceps and iridectomy scissors paying special attention to avoid damage of the aortic wall. 1-mm aortic ring slices were sectioned and embedded in a rat tail interstitial collagen gel (1.5 mg/mL) prepared by mixing 7.5 volumes of 2 mg/mL collagen, 1 volume of 10x HBBS, 1.5 volumes of 186 mM NaHCO_3_ and 0.1 volumes of 1 M NaOH to adjust the pH to 7.4. The collagen gels containing the aortic rings were polymerized in cylindrical agarose wells and kept in triplicate at 37°C in 60 mm diameter Petri dishes (bacteriological polysterene, Falcon, Becton Dickinson, Lincoln Park, New Yersey). Each Petri dish contained 6 mL of MCDB131 medium supplemented with 1% L-glutamine, 25 mM NaHCO_3_, 100 U/mL penicilin, 100 μg/mL streptomycin, in presence or absense of the tested antibodies. The cultures were kept at 37°C, 5% CO_2_ in a humidified environment, and photographed after 14 days. The antiangiogenic response was quantified by microvessel counting according to published criteria [45]. Experimental procedures with animals were conducted in accordance with the Spanish Legislation (Real Decreto 53/2013, BOE, 34/-11421, 2013) in compliance with the European Community Directive 2010/63/EU regulating the use and care of laboratory animals. The protocols were approved by the Ethics Committee for Animal Experiments of the University of Málaga.

### *In vivo* mouse matrigel plug assay

C57BL/6 female mice were injected s.c. near the abdominal midline, via a 23-gauge needle with 300 mL of Matrigel (Corning) containing FGF2 (1 μg/mL) and anti-SOD3 antibody (Abcam ab80946, mouse monoclonal) at 1/200 dilution. Positive control mice received the same volume of Matrigel with FGF2 mixed with the same amount of isotype control (IgG1, Abcam, Isotype Control ab91353). Negative control mice were injected with Matrigel containing the corresponding dose of PBS and IgG1. After injection, the Matrigel rapidly formed a single, solid gel plug. After 7 days, mice were sacrified and plugs were removed. Plugs were fixed in formalin solution (SIGMA), dehydrated and processed for paraffin embeding. Sections of 12 μm thickness were collected on poly-L-lysinated slides. Immunodetection of endomucin (endothelial marker; sc-65495, Santa Cruz Biotechnology, Inc, 1/100 dilution) and DAPI staining (1/1000 dilution) were performed and a minimum of 6 random fields of the sections were photographed under fluorescence microscope at 10X magnification. Endomucin and DAPI positive areas were quantified using ImageJ software and all data was expressed as means ± SD of 3 plugs (IgG) or 4 plugs (rest of conditions) and normalized to the IgG+FGF2 control (100%). Experimental procedures with animals were conducted in accordance with the Spanish Legislation (Real Decreto 53/2013, BOE, 34/-11421, 2013) in compliance with the European Community Directive 2010/63/EU regulating the use and care of laboratory animals. The protocols were approved by the Ethics Committee for Animal Experiments of the University of Málaga.

### Statistical analysis

Data shown are means ± standard deviation (SD) of at least three independent experiments. Statistical significance was determined by Student´s paired sample test. Values of p <0.05 were considered to be significant.

## SUPPLEMENTARY MATERIALS TABLE




