# Supplementary material for: In silico prediction of targets for anti-angiogenesis and their in vitro evaluation confirm the involvement of SOD3 in angiogenesis
Source: Oncotarget. 2018 Apr 3;9(25):17349–67. doi: 10.18632/oncotarget.24693 (PMC5915121; doi:10.18632/oncotarget.24693)
Supplement: Supplementary file 1 [file oncotarget-09-17349-s001.pdf]

In silico prediction of targets for anti-angiogenesis and their *in vitro* evaluation confirm the involvement of SOD3 in angiogenesis

SUPPLEMENTARY MATERIALS

Supplementary Table 1: DAVID Functional Annotation Table for SOD3.

See Supplementary File 1
