# Supplementary material for: In silico prediction of targets for anti-angiogenesis and their in vitro evaluation confirm the involvement of SOD3 in angiogenesis
Source: Oncotarget. 2018 Apr 3;9(25):17349–67. doi: 10.18632/oncotarget.24693 (PMC5915121; doi:10.18632/oncotarget.24693)
Supplement: Supplementary file 2 [file oncotarget-09-17349-s002.pdf]

SOD3	Superoxide dismutase	Related Genes	Xanthomonas campestris
COG_ONTOLOGY	Inorganic ion transport and metabolism,		
GOTERM_BP_FAT	oxygen and reactive oxygen species metabolic process, superoxide metabolic process, oxidation reduction,		
GOTERM_MF_FAT	superoxide dismutase activity, antioxidant activity, oxidoreductase activity, acting on superoxide radicals as acceptor, ion binding, cation binding, metal ion binding,		
INTERPRO	Manganese/iron superoxide dismutase, Manganese/iron superoxide dismutase, N-terminal, Manganese/iron superoxide dismutase, C-terminal, Manganese/iron superoxide dismutase, binding site,		
PIR_SUPERFAMILY	PIRSF000349:SODismutase,		
SP_PIR_KEYWORDS	complete proteome, oxidoreductase,		
SOD3	Superoxide dismutase [Cu-Zn]	Related Genes	Anopheles gambiae
GOTERM_BP_FAT	oxygen and reactive oxygen species metabolic process, superoxide metabolic process, oxidation reduction,		
GOTERM_MF_FAT	superoxide dismutase activity, copper ion binding, zinc ion binding, antioxidant activity, oxidoreductase activity, acting on superoxide radicals as acceptor, ion binding, cation binding, metal ion binding, transition metal ion binding,		
INTERPRO	Superoxide dismutase, copper/zinc binding, Superoxide dismutase, copper/zinc, binding site,		
PIR_SUPERFAMILY	PIRSF000348:superoxide dismutase [Cu-Zn],		
SP_PIR_KEYWORDS	complete proteome, copper, metal-binding, oxidoreductase, zinc,		
SOD3	hypothetical protein LOC100127868	Related Genes	Xenopus (Silurana) tropicalis
GOTERM_BP_FAT	oxygen and reactive oxygen species metabolic process, superoxide metabolic process, oxidation reduction,		
GOTERM_MF_FAT	superoxide dismutase activity, copper ion binding, zinc ion binding, antioxidant activity, oxidoreductase activity, acting on superoxide radicals as acceptor, ion binding, cation binding, metal ion binding, transition metal ion binding,		
INTERPRO	Superoxide dismutase, copper/zinc binding, Superoxide dismutase, copper/zinc, binding site,		
SP_PIR_KEYWORDS	copper, metal-binding, oxidoreductase, zinc,		
SOD3	similar to extracellular-superoxide dismutase (EC 1.15.1.1); superoxide dismutase 3, extracellular	Related Genes	Gallus gallus
GOTERM_BP_FAT	oxygen and reactive oxygen species metabolic process, superoxide metabolic process, oxidation reduction,		
GOTERM_MF_FAT	ion binding, cation binding, metal ion binding,		
SOD3	superoxide dismutase 3, extracellular	Related Genes	Bos taurus
GOTERM_BP_FAT	response to hypoxia, oxygen and reactive oxygen species metabolic process, superoxide metabolic process, oxidation reduction, response to oxygen levels,		
GOTERM_CC_FAT	extracellular region, extracellular space, Golgi apparatus, trans-Golgi network, extracellular region part, Golgi apparatus part,		
GOTERM_MF_FAT	superoxide dismutase activity, copper ion binding, zinc ion binding, antioxidant activity, oxidoreductase activity, acting on superoxide radicals as acceptor, ion binding, cation binding, metal ion binding, transition metal ion binding,		
INTERPRO	Superoxide dismutase, copper/zinc binding, Superoxide dismutase, copper/zinc, binding site,		
SP_PIR_KEYWORDS	copper, metal-binding, oxidoreductase, zinc,		
SOD3	superoxide dismutase 3, extracellular	Related Genes	Rattus norvegicus
GOTERM_BP_FAT	response to reactive oxygen species, response to superoxide, response to oxygen radical, response to hypoxia, oxygen and reactive oxygen species metabolic process, superoxide metabolic process, response to oxidative stress, response to inorganic substance, response to metal ion, response to copper ion, oxidation reduction, response to oxygen levels,		
GOTERM_CC_FAT	extracellular region, extracellular space, Golgi apparatus, trans-Golgi network, extracellular matrix, extracellular region part, Golgi apparatus part,		
GOTERM_MF_FAT	superoxide dismutase activity, copper ion binding, zinc ion binding, antioxidant activity, oxidoreductase activity, acting on superoxide radicals as acceptor, ion binding, cation binding, metal ion binding, transition metal ion binding,		
INTERPRO	Superoxide dismutase, copper/zinc binding, Superoxide dismutase, copper/zinc, binding site,		
PIR_SUPERFAMILY	PIRSF000348:superoxide dismutase [Cu-Zn],		
SP_PIR_KEYWORDS	antioxidant, copper, disulfide bond, glycoprotein, metal-binding, metalloprotein, oxidoreductase, Secreted, signal, zinc,		
UP_SEQ_FEATURE	chain:Extracellular superoxide dismutase [Cu- Zn], disulfide bond, glycosylation site:N-linked (GlcNAc...), metal ion-binding site:Copper; catalytic, metal ion-binding site:Zinc; structural, mutagenesis site, sequence conflict, signal peptide,		
SOD3	superoxide dismutase 3, extracellular	Related Genes	Mus musculus
GOTERM_BP_FAT	response to hypoxia, oxygen and reactive oxygen species metabolic process, superoxide metabolic process, oxidation reduction, response to oxygen levels,		
GOTERM_CC_FAT	extracellular region, extracellular space, Golgi apparatus, trans-Golgi network, extracellular region part, Golgi apparatus part,		
GOTERM_MF_FAT	superoxide dismutase activity, copper ion binding, zinc ion binding, antioxidant activity, oxidoreductase activity, acting on superoxide radicals as acceptor, ion binding, cation binding, metal ion binding, transition metal ion binding,		
INTERPRO	Superoxide dismutase, copper/zinc binding, Superoxide dismutase, copper/zinc, binding site,		
PIR_SUPERFAMILY	PIRSF000348:superoxide dismutase [Cu-Zn],		
SP_PIR_KEYWORDS	3d-structure, antioxidant, copper, direct protein sequencing, disulfide bond, glycoprotein, metal-binding, oxidoreductase, Secreted, signal, zinc,		
UP_SEQ_FEATURE	chain:Extracellular superoxide dismutase [Cu- Zn], disulfide bond, glycosylation site:N-linked (GlcNAc...), metal ion-binding site:Copper; catalytic, metal ion-binding site:Zinc; structural, signal peptide,		
SOD3	superoxide dismutase 3, extracellular	Related Genes	Oryctolagus cuniculus
GOTERM_BP_FAT	response to hypoxia, oxygen and reactive oxygen species metabolic process, superoxide metabolic process, oxidation reduction, response to oxygen levels,		
GOTERM_CC_FAT	extracellular region, extracellular space, Golgi apparatus, trans-Golgi network, extracellular region part, Golgi apparatus part,		
GOTERM_MF_FAT	superoxide dismutase activity, copper ion binding, zinc ion binding, antioxidant activity, oxidoreductase activity, acting on superoxide radicals as acceptor, ion binding, cation binding, metal ion binding, transition metal ion binding,		

INTERPRO	Superoxide dismutase, copper/zinc binding, Superoxide dismutase, copper/zinc, binding site,		
PIR_SUPERFAMILY	PIRSF000348:superoxide dismutase [Cu-Zn],		
SP_PIR_KEYWORDS	antioxidant, copper, disulfide bond, glycoprotein, metal-binding, oxidoreductase, Secreted, signal, zinc,		
UP_SEQ_FEATURE	chain:Extracellular superoxide dismutase [Cu- Zn], disulfide bond, glycosylation site:N-linked (GlcNAc...), metal ion-binding site:Copper; catalytic, metal ion-binding site:Zinc; structural, sequence conflict, signal peptide,		
**SOD3**	**superoxide dismutase 3, extracellular**	**Related Genes**	**Homo sapiens**
BIOCARTA	The IGF-1 Receptor and Longevity,		
GOTERM_BP_FAT	response to hypoxia, oxygen and reactive oxygen species metabolic process, superoxide metabolic process, response to inorganic substance, response to metal ion, response to copper ion, oxidation reduction, response to oxygen levels,		
GOTERM_CC_FAT	cell fraction, extracellular region, extracellular space, soluble fraction, Golgi apparatus, trans-Golgi network, extracellular matrix, extracellular region part, Golgi apparatus part,		
GOTERM_MF_FAT	pattern binding, superoxide dismutase activity, copper ion binding, glycosaminoglycan binding, heparin binding, zinc ion binding, antioxidant activity, oxidoreductase activity, acting on superoxide radicals as acceptor, carbohydrate binding, polysaccharide binding, ion binding, cation binding, metal ion binding, transition metal ion binding,		
INTERPRO	Superoxide dismutase, copper/zinc binding, Superoxide dismutase, copper/zinc, binding site,		
OMIM_DISEASE	Superoxide dismutase, elevated extracellular,		
PIR_SUPERFAMILY	PIRSF000348:superoxide dismutase [Cu-Zn],		
SP_PIR_KEYWORDS	3d-structure, antioxidant, complete proteome, copper, direct protein sequencing, disulfide bond, glycation, glycoprotein, heparin-binding, metal-binding, metalloprotein, oxidoreductase, polymorphism, Secreted, signal, zinc,		
UP_SEQ_FEATURE	chain:Extracellular superoxide dismutase [Cu- Zn], disulfide bond, glycosylation site:N-linked (Glc) (glycation); in vitro, glycosylation site:N-linked (GlcNAc...), helix, metal ion-binding site:Copper; catalytic, metal ion-binding site:Zinc; structural, sequence variant, signal peptide, site:Not glycated, strand,		
**SOD3**	**superoxide dismutase3**	**Related Genes**	**Zea mays**
COG_ONTOLOGY	Inorganic ion transport and metabolism,		
GOTERM_BP_FAT	oxygen and reactive oxygen species metabolic process, superoxide metabolic process, oxidation reduction,		
GOTERM_CC_FAT	mitochondrion, mitochondrial matrix, membrane-enclosed lumen, mitochondrial lumen, organelle lumen, mitochondrial part, intracellular organelle lumen,		
GOTERM_MF_FAT	superoxide dismutase activity, antioxidant activity, oxidoreductase activity, acting on superoxide radicals as acceptor, manganese ion binding, ion binding, cation binding, metal ion binding, transition metal ion binding,		
INTERPRO	Manganese/iron superoxide dismutase, Manganese/iron superoxide dismutase, N-terminal, Manganese/iron superoxide dismutase, C-terminal, Manganese/iron superoxide dismutase, binding site,		
PIR_SUPERFAMILY	PIRSF000349:SODismutase, PIRSF000349:superoxide dismutase, Mn/Fe specific,		
SP_PIR_KEYWORDS	manganese, metal-binding, mitochondrion, oxidoreductase, transit peptide,		
UP_SEQ_FEATURE	chain:Superoxide dismutase [Mn] 3.1, mitochondrial, metal ion-binding site:Manganese, transit peptide:Mitochondrion,		

Please cite *Nature Protocols 2009; 4(1):44 & Genome Biology 2003; 4(5):P3* within any publication that makes use of any methods inspired by **DAVID**.
